# A sequential explanatory mixed-methods study of UK higher education teacher experiences when building rapport with international students online

**DOI:** 10.1007/s43545-022-00533-2

**Published:** 2022-09-28

**Authors:** Gemma Peacock

**Affiliations:** University of Reading, Reading, UK

**Keywords:** Rapport, Teacher student relationships, Online learning environments, Emergency remote teaching, Covid-19

## Abstract

Effective, interpersonal teacher–student relationships and positive rapport are widely known to positively impact student attainment and learning experiences. Establishing and maintaining these have been particularly challenging, however, since the Covid-19 pandemic forced higher education (HE) teaching online in 2020. This study, therefore, aimed to explore English for Academic Purposes (EAP) teachers’ experiences of building rapport with international students in online learning environments in UK HE institutions during emergency remote teaching via sequential explanatory mixed methods and three research questions. Study Phase 1 comprised a structured, online, quantitative and qualitative questionnaire completed by 36 participants of the target population from 19 UK HE institutions, while Phase 2 employed in-depth qualitative interviews with three selected participants from Phase 1. Three themes emerged as most significant when considering teacher experiences of building rapport with students online: online communication in online sessions; teacher–student communication outside online sessions; and teacher availability and accessibility. The findings suggest that if online sessions could primarily be used as opportunities to meet learning outcomes, then the necessary and conscious task of building teacher–student rapport might be more effective in frequent 1–1 tutorials with students or during any available pre- and post-online session time.

## Introduction

In March 2020, teaching in all UK sectors was forced online due to the Covid-19 pandemic (World Health Organization n.d) and the related national lock-down. In the higher education (HE) sector, English for Academic Purposes (EAP) face-to-face (F2F) courses were rapidly converted and moved online, where in most cases no online course provision had previously existed. Pedagogically and technically unprepared university teachers found themselves delivering online courses from home instead of teaching in the classroom. This became known as emergency remote teaching (Hodges et al. 2020). Beyond the immediate challenges of the new technical, administrative and practical aspects of online teaching, these teachers also experienced challenges establishing and maintaining positive teacher–student relationships with their international students in the online learning environment (OLE).

Achievement of learning outcomes and positive student experiences can be supported by effective interpersonal teacher–student relationships (Cuseo 2018; Glazier 2016; Mseleku 2020; Parnes et al. 2020; Shudde 2019) both in F2F instruction and in the online learning environment (see Boling et al. 2012; Buttler et al. 2021). In fact, how successfully a teacher builds positive rapport with their students can not only impact academic performance (Frisby and Martin 2010), but also student attrition rates and course engagement levels (Benson et al. 2005), as well as student motivation and well-being (Granitz et al. 2009; Shim and Song 2020). In the specific context of second-language learning such as EAP, positive teacher–student rapport (TSR) can contribute to a positive learning environment, which in turn can positively affect students’ language development (Steinberg et al. 2001). This is particularly important for international EAP students since teaching and learning EAP is a teacher–student collaboration where both bring specific knowledge to the learning environment (Alexander et al. 2008).

International students have specific needs in online learning environments due to their diverse educational and ethno-linguistic backgrounds, and require positive relationships with their teachers to develop cross-cultural communication skills needed to assimilate in the UK HE environment in their future courses (Alfattal 2016; Estepp and Roberts 2015). While a body of literature giving a snapshot of pedagogy at this time is emerging, it is by no means extensive therefore more research is needed to reach consensus on the unprecedented pedagogical shift which took place and to start to identify best practices in the OLE.

This study explored EAP teachers’ experiences of building rapport with international students in the OLE in UK HE institutions between March and September 2020 of the Covid-19 pandemic. It built on Murphy and Rodríguez-Manzanares’ (2012) research, in which they provided insights into the importance of TSR in distance education and established categories and indicators of rapport based on their summary of the literature and their own research. Compared to Murphy and Rodríguez-Manzanares’ (2012) distance education context, online teaching and learning today generally includes greater teacher–student and student–student interaction, as well as more live ‘in-person’ interaction via video calling technology. This is due to the significant advancements in technology and online pedagogy made since 2012. Despite these differences, the overlap between distance education and modern online teaching and learning is large enough to justify using Murphy and Rodríguez-Manzanares’ research as a springboard for investigation into this topic, particularly in a domain such as rapport with limited literature in the online teaching context.

For the purposes of this study the term ‘online learning environment’ (OLE) is used to describe both synchronous and asynchronous learning tasks and interactions, occurring on an institution’s virtual learning environment (VLE), via independent technology, tools or applications such as Zoom™, Microsoft Teams™ or simply on the World Wide Web. The term ‘online session’ refers to a synchronous online class using video conferencing software.

This study addressed a significant gap in EAP research and in online rapport-building research specifically. While extensive literature exists on a wide range of aspects of distance learning and online learning pre-Covid-19 (see Means et al. 2009), there is limited research into online learning post-Covid-19 due to its recency. The literature refers to the need for a thorough examination of rapport and teacher–student relationships in novel environments such as the online learning space (Glazier 2016; Sidelinger et al. 2016; Webb and Barrett 2014), particularly following the Covid-19 pandemic (Buttler et al. 2021). The earliest studies on rapport in F2F educational contexts appeared in the early 2000s (Faranda and Clarke 2004) but the literature on rapport is mostly situated in the fields of psychology (Bernieri et al. 1996; Miles et al. 2009), medicine (O’Connor 2005), counselling (Sharpley et al. 2006) and business (Macintosh 2009) where building rapport in-person rather than online has been the focus. Likewise in the field of education, there is limited examination of rapport in the OLE (Glazier 2016; Kaufmann and Vallade 2020; Rolfe 2015; Vos 2015) compared to studies focusing on rapport in F2F learning environments. Finally, the complex phenomenon of rapport in teacher–student relationships is an under-researched area in comparison to the literature on rapport in student–student relationships (Kaufmann and Vallade 2020; Lammers et al. 2017; Sailor 2020; Tinto 2012) particularly in the specific case of EAP and international students who arguably have different needs to home students (Alfattal 2016; Misra et al. 2003). For these reasons this study has value since it addresses these research gaps.

This study has particular value since online teaching in some form is likely to continue across multiple education sectors, due to the continuance of the Covid-19 pandemic and the rapid development of online pedagogical theory, online teaching modalities and learning technologies. Covid-19 has had significant implications on international student numbers and has severely impacted the income on which academic institutions rely to fund research, investment and widening participation activities (Bolton and Hubble 2021). It is therefore critical to investigate the ways in which online learning can be leveraged for successful learning outcomes and student experiences for international students, to ensure their association with UK HE institutions. While the focus of this study is the EAP context, rapport itself is born out of human interaction in any environment; therefore the findings may also be valuable to other online educational and non-educational contexts.

Based on the context and research gaps identified above this study seeks to answer one overarching research question: *What were EAP teachers’ experiences of building rapport with international students in online learning environments in UK higher education (HE) institutions in the early Covid-19 era?* This broad question will be investigated through three research sub-questions (RQ):

RQ1—What are EAP teachers’ perceptions of the importance of various categories of rapport when building teacher–student rapport in synchronous and asynchronous online learning environments?

RQ2—What perceived constraints and challenges were reported by teachers when building teacher–student rapport online?

RQ3—Which online teaching practices and student-directed rapport behaviours did teachers employ to help develop teacher–student rapport online?

## Literature review

### Background

#### The field of EAP

English for Academic Purposes within HE has been defined as the “the study of language in academic contexts” (Bruce 2011, p.116) aiming to support learners “to study, conduct research or teach” in English (Flowerdew and Peacock 2001a, p.8). It is a branch of English for Specific Purposes (ESP), which, alongside English for General Purposes (EGP), comprises the two distinct branches of English Language Teaching (ELT) (Flowerdew and Peacock 2001b).

EAP programmes serve to equip international students with the language skills necessary for academic study in English and knowledge of academic culture and practices. Before 2020, such courses were typically taught F2F. EAP students not only require academic study skills development and English language knowledge (both general academic and discipline-specific vocabulary) in order to engage with academic texts in their discipline but they also require disciplinary specific knowledge.

### The construct of rapport

#### Definitions of rapport

To answer the research sub-questions, the construct of rapport must firstly be defined. Rapport is a complex and somewhat nebulous concept. Carey et al. (1986) define it as a relationship with mutual understanding and satisfactory communication. Hall et al. (2009, p. 324) define it in more detail as “a relationship that [is] pleasant and engaging, a high degree of liking or positive affect, mutual attention, harmonious relation, easy/smooth communication and/or symmetry and synchrony in the interaction”. Tickle-Degnen and Rosenthal (1990) describe mutual attentiveness, positivity and coordination as three interrelated components exhibited through verbal and non-verbal behaviours. In the educational context Bernieri (1988, p. 120) describes rapport as “harmonious interactions” between teachers and students.

Rapport is only experienced in dyadic interactions, between two individuals such as teacher and student (Altman 1990), therefore it cannot be defined as a personality trait (Tickle-Degnen and Rosenthal 1990). Instead, it distinguishes itself as a phenomenon of ‘mutuals’: attentiveness (Tickle-Degnen and Rosenthal 1990), respect (Kyriacou 2009), openness (Granitz et al. 2009), positive attention (Hall et al. 2009) and understanding (Carey et al. 1986). When attention is negative, rapport cannot exist (Tickle-Degnen and Rosenthal 1990) therefore harmony is one of its critical elements: in harmonious understanding (Kyriacou 2009), interactions (Bernieri 1988) and relations (Gremler and Gwinner 2000). Rapport is also a dynamic and evolving process, relying on collaboration between teacher and student to enable growth and learning (Rogers 2012; Spencer-Oatey 2000).

#### Indicators and categories of rapport

According to the literature, rapport can manifest in a variety of ways in F2F environments. Despite limited research on this in the OLE, it is necessary to identify these since RQ1 investigates teacher perceptions of the importance of various categories of rapport, and RQ3 investigates the ways in which teachers chose to build rapport with their students online.

Physical ways in which rapport manifests include coordinated movement (Bernieri 1988) or body language (Marks 1994); matching voice tone or gestures (Nickels et al. 1983); the mirroring of behaviour or posture (Granitz et al. 2009; Lakin and Chartrand 2003). Tickle-Degnen and Rosenthal (1990) focused on nonverbal behaviour as a tool for creating rapport between individuals which focused on posture, gesture and interactional synchrony. Rapport is also seen through ‘mutual respect’ in effective classrooms which, for teachers, manifests as awareness of student needs, progress monitoring, helping patiently, giving praise and showing concern (Kyriacou 2009).

Murphy and Rodríguez-Manzanares (2012) placed rapport indicators articulated in the general literature into eight categories: disclosure, honesty and respect; supporting and monitoring; recognising the person/individual; sharing, mirroring, mimicking, matching; interacting socially; availability, accessibility and responsiveness; caring and bonding; communicating effectively. These categories are referred to in Phase 1 of this study in order to answer RQ1. Murphy and Rodríguez-Manzanares (2012) also identified six major categories of rapport-building in both synchronous and asynchronous distance education which are applicable to the online learning environment. These are: recognising the person/individual; supporting and monitoring; availability, accessibility and responsiveness; non text-based interactions; tone of interactions; non-academic conversation or interactions. See Table 1 for the sub-categories given for each particular category. These categories are referred to in Phase 1 of this study in order to answer RQ3. In OLEs, most of these categories and indicators of rapport still apply although physical manifestations of rapport ultimately depend on the availability of visual cues and physical presence via web-camera. These categories and indicators of research were employed in this research since they offered a means by which to measure rapport, and they were derived from interviews with 42 Canadian high-school distance education teachers which is relatable to the focus of this study.

**Table 1:** Summary of distance education rapport categories and subcategories (Murphy and Rodríguez-Manzanares 2012)

Categories	Sub-categories
Recognising the person/individual	Eliciting personal information
	Expressing personality
	Acknowledging the person
Supporting and monitoring	Supporting and monitoring
	Praising
	Providing feedback
Availability, accessibility, and responsiveness	Being available
	Responding quickly
Non-text-based interactions	Hearing each other
	Seeing each other
	Interacting in real-time, face-to-face
Tone of interactions	Being friendly
	Being humorous
	Being respectful and honest
Non-academic conversation/interactions	Conversing socially
	Showing care and concern

## Methodology

### Research design

This study employed the third *research paradigm* (Kuhn 1962) of mixed methods sequential explanatory research design to collect cross-sectional data. Both quantitative and qualitative data were collected in the research questionnaire in Phase 1 and then more in-depth qualitative data were collected via interviews in Phase 2.

Quantitative research allowed for standardised data collection and statistical analysis in order to answer RQ1 and to gather initial information for RQ3 (Table 2). The strengths of using quantitative methods for these parts of the study include the fact that such results are structured, measurable, objective and focused on causation (Bryman 2015). Qualitative data collection methods were employed to answer RQ2 (in both Phase 1 and 2) and to expand on the quantitative data previously collected on RQ3 (Table 2). They increase understanding by exposing connections between participant experiences and interactions with certain phenomena (Croker 2009). They also align with a social constructivist perspective where knowledge is constructed by groups aiming to understand complex lived experiences (Richards 2003). Qualitative research was therefore a suitable joint research method for this study since the ultimate aim was to explore teacher experiences with building teacher–student rapport online rather than solely generating statistics. Qualitative research is by nature “exploratory” (Croker 2009, p. 7) which suits a research area with limited research to date.

**Table 2:** Research sub-question, study phase, data type and collection method

Research sub-question number	Research sub-question	Study phase and data type	Data collection method
RQ1	What are EAP teachers’ perceptions of the importance of various categories of rapport when building teacher–student rapport in synchronous and asynchronous online learning environments?	Phase 1—quantitative	Research questionnaire—Sect. 2 (ranking question)
RQ2	What perceived constraints and challenges were reported by teachers when building teacher–student rapport online?	Phase 1—Qualitative	Research questionnaire—Sect. 3 (typed comments)
		Phase 2—Qualitative	Interview
RQ3	Which online teaching practices and student-directed rapport behaviours did teachers employ to help develop teacher–student rapport online?	Phase 1—Quantitative	Research questionnaire—Sect. 3 (Likert-type scale responses)
		Phase 2—Qualitative	Interview

### Recruitment of participants and ethics procedures

The recruitment of participants for this study employed “typical sampling” representing a target sample of the target population selected according to the research focus (Dornyei 2007, p. 128). EAP teachers who taught on UK HE online EAP courses synchronously and asynchronously between March and September 2020 were recruited via an email request for participants via the BALEAP online mailing list (the Global Forum for EAP Practitioners). Such teachers were also sourced from the researcher’s colleagues at the University of Reading. All teacher participants in this study held British nationality.

Since a fundamental principle of scientific inquiry is ensuring an ethically sound research process (see Kjellström et al. 2010), ethics clearance was granted by the University of Portsmouth. Ethical principles relating to the respect of study participants were closely followed during the study. These included gaining appropriately informed consent from participants regarding the use of their data. Participation in the study was entirely voluntary with the right to withdraw in person and to withdraw data up until the point of data analysis. Data were anonymised and securely stored to assure confidentiality. The nature of the research was fully explained to participants through an information sheet.

### Phase 1: research questionnaire

The first phase of the study comprised a self-developed, structured, online, quantitative and qualitative questionnaire completed by 36 participants of the target population (EAP teachers from 19 UK HE institutions who taught online asynchronously and synchronously from March to September 2002). This was an appropriate sample size due to time and resource limitations and since the method of data analysis chosen was descriptive statistics for which “nearly any sample size will suffice” (Israel 1992, p. 4). The questionnaire comprised three logical sections with their own distinct theme to facilitate clarity. To ensure the quality of the responses and thus of the survey data overall, the design of the research questionnaire was carefully considered within the Total Survey Error framework (Groves et al. 2009).

Section one of the questionnaire gathered quantitative demographic and background information from participants according to various quantitative and categorical variables. Such data are useful for results analysis, generalisation of findings and comparison alongside other data sets (APA 1994).

Section two of the questionnaire directly related to RQ1 and, via two quantitative ranking questions, explored teacher perceptions of the importance of two different sets of categories of rapport when building rapport with students in both asynchronous and synchronous online learning environments. Question 13 involved the ranking of eight categories of rapport which were posited by Murphy and Rodríguez-Manzanares (2012) after their review of both general and distance education literature. Question 14 involved the ranking of six further categories of rapport which were identified by the same researchers after interviewing 42 teachers in their own 2012 study. One of Murphy and Rodríguez-Manzanares’ original study objectives was to identify teachers’ perceived importance of rapport; however their question design did not allow for meaningful data collection. Questions 13 and 14 therefore expanded on those original limited findings by asking teachers to separately rank the two rapport category sets to gather more detailed data on which particular aspects of rapport-building EAP teachers perceived to be the most important in the online teaching and learning environment.

Ranking questions were used in section two since they allowed for the direct comparison of different items and they ensured that a different value was ascribed to each item, enabling the question to be answered clearly. One negative aspect of such questions, however, is that forcing respondents to choose between items that may be of the equivalent value to them (Krosnick 1999) may negatively affect the reliability and validity of the results. Keeping ranking items below eight in number mitigated the fact that ranking questions take longer to complete than other question types (Munson and McIntyre 1979) and are more cognitively challenging to answer (Krosnick 1991).

Section three of the questionnaire collected quantitative data for RQ3 and qualitative data for RQ2. Individual Likert-type items were used for the purpose of individual analysis, rather than for the formation of composite scales in the data analysis stage (Clason and Dormody 1994) since quantitative measures of teacher characteristics were not the focus of this study. Likert-type questions are appropriate for measuring teacher perceptions and beliefs and they can facilitate data analysis (Likert 1932). Section three used fourteen five-point Likert-type questions and optional text comment boxes for respondents to type reasons for their answer when a neutral or negative response was given to a question. These questions were derived directly from Murphy and Rodríguez-Manzanares’ (2012) sub-categories of rapport in distance education.

Finally, the overall questionnaire was designed to minimise bias. Ranking and rating questions were chosen instead of agree-disagree question types in order to minimise the acquiescence response bias. Anonymising survey responses should result in minimised social desirability bias. The pilot survey and subsequent adjustments minimised other identified questionnaire biases.

#### Quantitative data analysis procedures

Descriptive statistics methods were used for quantitative data analysis in both section two and section three of the questionnaire to facilitate data interpretation and observation of data trends. Descriptive statistics for ranking data (such as that in section two of the questionnaire) aim to show the central tendency of respondents’ answers therefore two statistical methods were employed:

1. Frequency distribution—How many times was an option ranked at a particular value?
2. Mean rank (weighted average ranking)—What was the most popular choice?

Descriptive statistics for ordinal measurement scale items (such as the Likert-type questions in section three of the questionnaire) included frequency distribution and the mode (most common result per item) to illustrate the central tendency of the data.

### Phase 2: interview

The second phase of the study employed formal, in-depth, semi-structured, forty minute online interviews via video call on Microsoft Teams to gather qualitative data. Phase 2 therefore aimed to refine and elaborate on the initial data gathered in section three of Phase 1 to answer RQ2 and RQ3 more fully:

RQ2: What perceived constraints and challenges were reported by teachers when building teacher–student rapport online?

RQ3: Which online teaching practices and student-directed rapport behaviours did teachers employ to help develop teacher–student rapport online?

Three participants from Phase 1 were selected for online interview based on features of their demographic information from questionnaire section one, such as age, gender, EAP teaching experience, online teaching experience, course duration, and type and number of students. This ensured a variety of responses in the interview, therefore giving a more “realistic picture” of the target population (Richards 2003). These interviews were recorded with the interviewees’ permission so that they could later be transcribed verbatim before data analysis.

#### Qualitative data analysis procedures

The qualitative data analysis stage employed an iterative, inductive coding process to produce a rich analysis of the themes within the data to answer the research sub-questions. Themes can be defined as “a pattern of shared meaning underpinned by a central concept or idea” (Braun et al. 2021, para.3).

First, this involved analysis of the respondents’ typed comments from Phase 1 for RQ2 (which influenced the interview questions). Then verbatim transcriptions of the recorded interviews from Phase 2 were analysed and initial descriptive codes assigned to “chunks of meaning” (Marshall 1981, p. 396) within both data sets. These two qualitative data sets were combined and analysed in a cyclical process and recoded to produce a list of defined and named themes and categories (Braun et al. 2021) according to the relationships between the data and any pattern of regularities (Patton 2002).

Bias was minimised during the qualitative data analysis by employing an inductive process in which data coding and development of common themes were driven by data content rather than existing concepts or the researcher’s own ideas (Strauss and Corbin 1998).

## Findings and discussion

### Phase 1 quantitative findings

In the first phase of the study 51 potential participants were contacted or contacted the researcher directly. Of these 51 potential participants, 36 completed the research questionnaire giving a response rate of 71%. Descriptive statistics have been used in this section to summarise the characteristics and responses of the target sample.

#### Questionnaire section one

Section one of the questionnaire collected the study participants’ demographic information. This included age, gender, EAP teaching experience, general online teaching experience, the course duration and type of student (foundation or post-graduate) during the study timeframe, the percentage of synchronous online learning activities in that course, the technology used for asynchronous and for synchronous learning activities, and the size of class.

#### Questionnaire section two

Section two contained two questions (Q13 and Q14) both of which gathered data to answer RQ1 *(What are EAP teachers’ perceptions of the importance of various categories of rapport when building teacher–student rapport in synchronous and asynchronous online learning environments?).* Since changes were made to this question in the pilot study, the internal validity of this question and its results should be high (van Teijlingen and Hundley 2001).

**Fig. 1:**
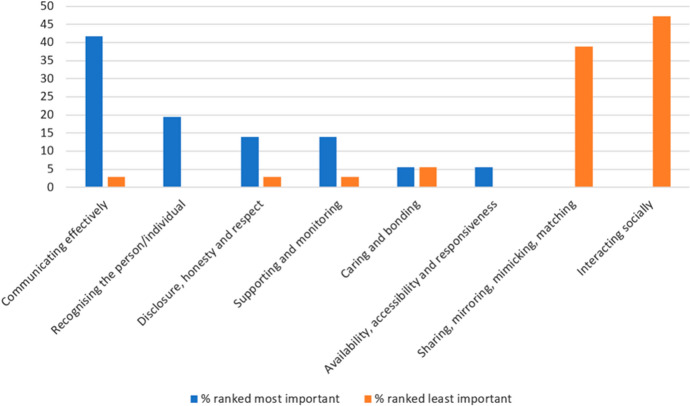
Percentage ranking eight rapport categories most important (value 1) and least important (value 8) (Q13)

Question 13 contained eight categories of rapport drawn from general and distance education literature which participants ranked in order of importance to them when teaching EAP online, to include both synchronous and asynchronous teaching and learning activities. Figure 1 shows the distribution of responses (marginal frequency) given for the most important and least important of the eight rapport categories when teaching online.

**Fig. 2:**
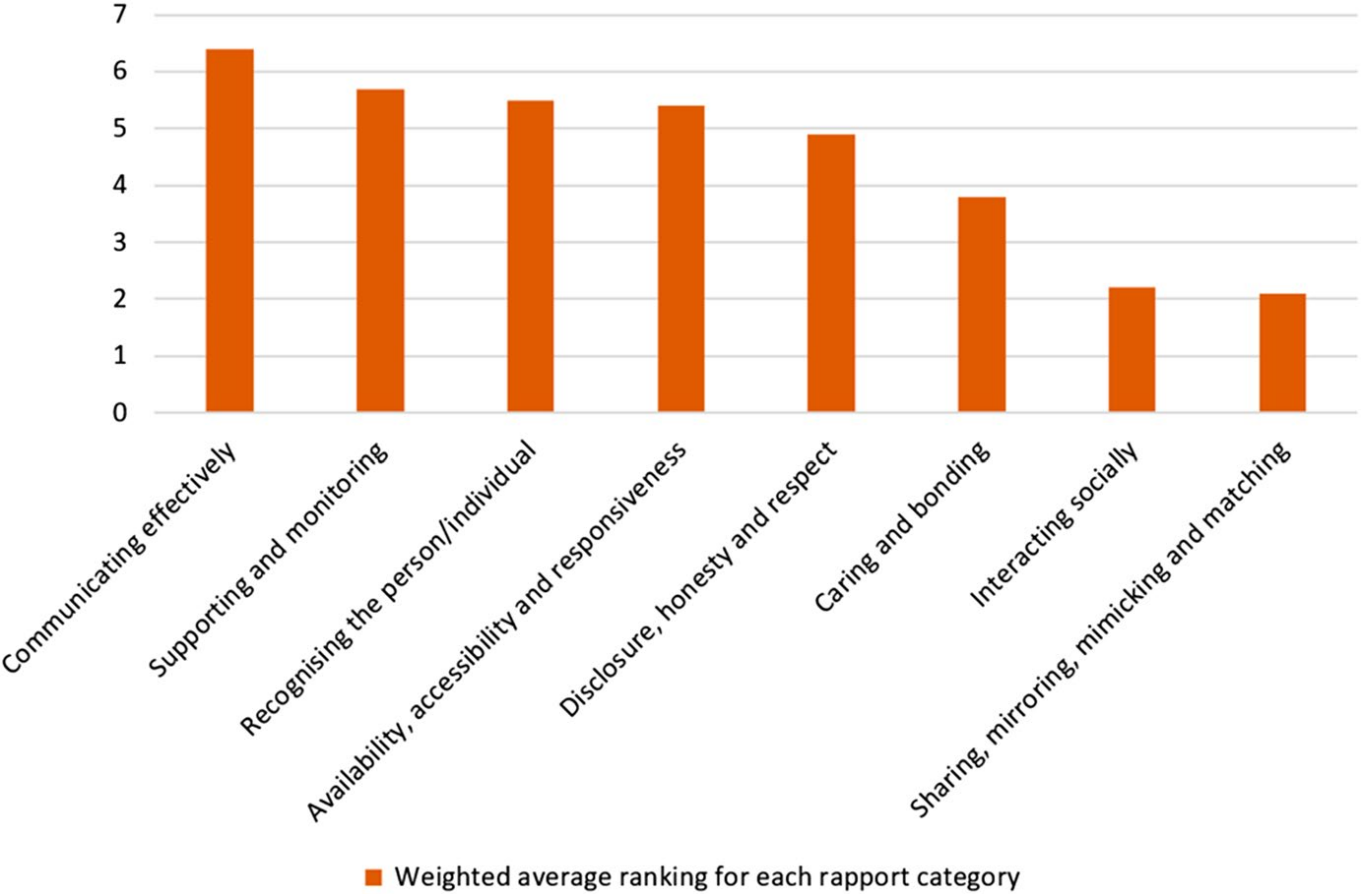
Weighted average ranking for rapport categories (8—most important, 1—least important) (Q13)

Figure 2 shows the weighted average ranking (mean) for each rapport category. This considers the varying importance of all the numbers in the data set (since Q13 of the questionnaire was a ranking question), therefore it is an efficient summary measure since it uses all available data. The weighted average data in Fig. 2 therefore reflect the relative importance of each rapport category according to the respondents. The overall trend here reflects that in Fig. 1 which indicates the reliability and validity of question 13.

**Fig. 3:**
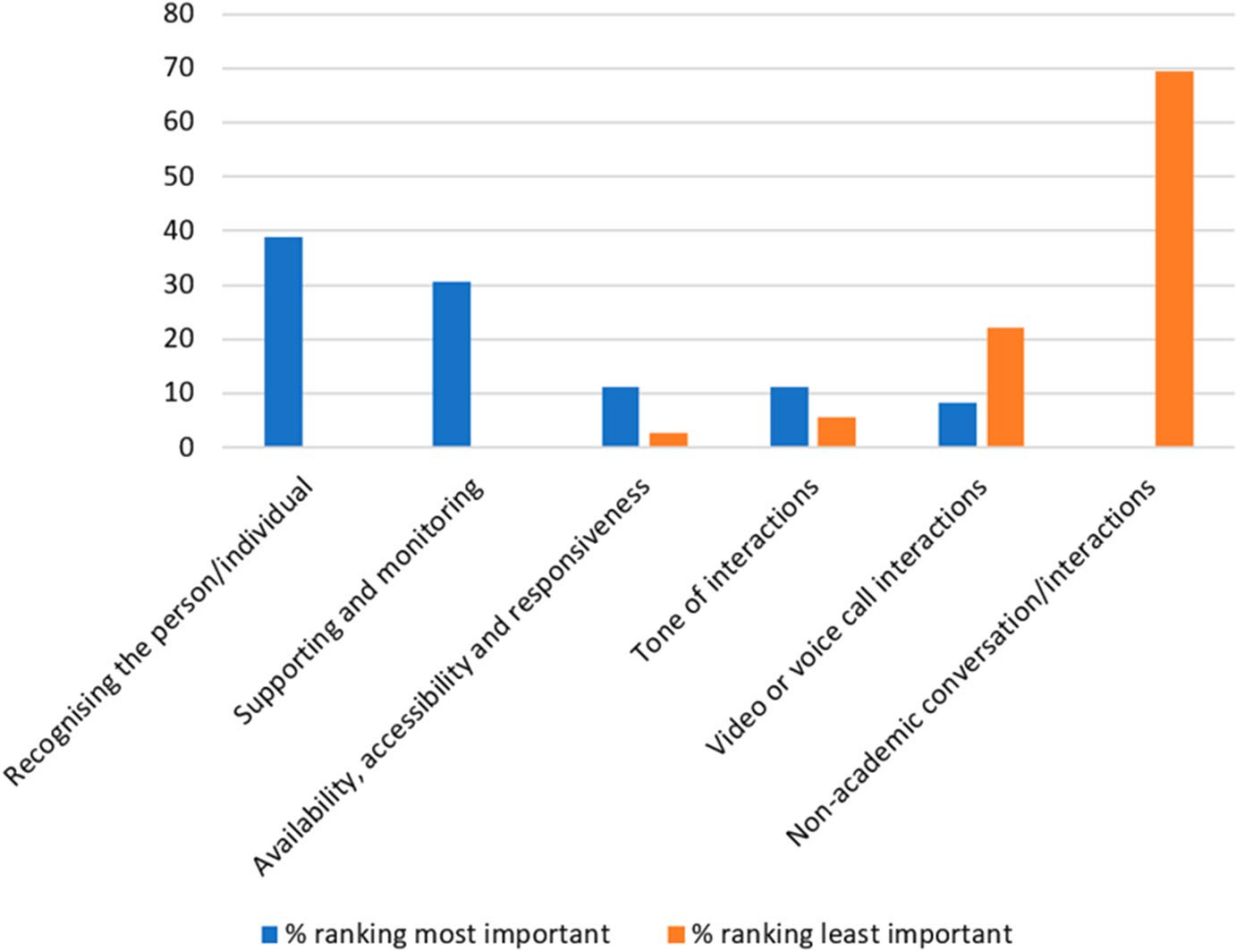
Percentage ranking six rapport categories most important (value 1) and least important (value 6) (Q14)

**Fig. 4:**
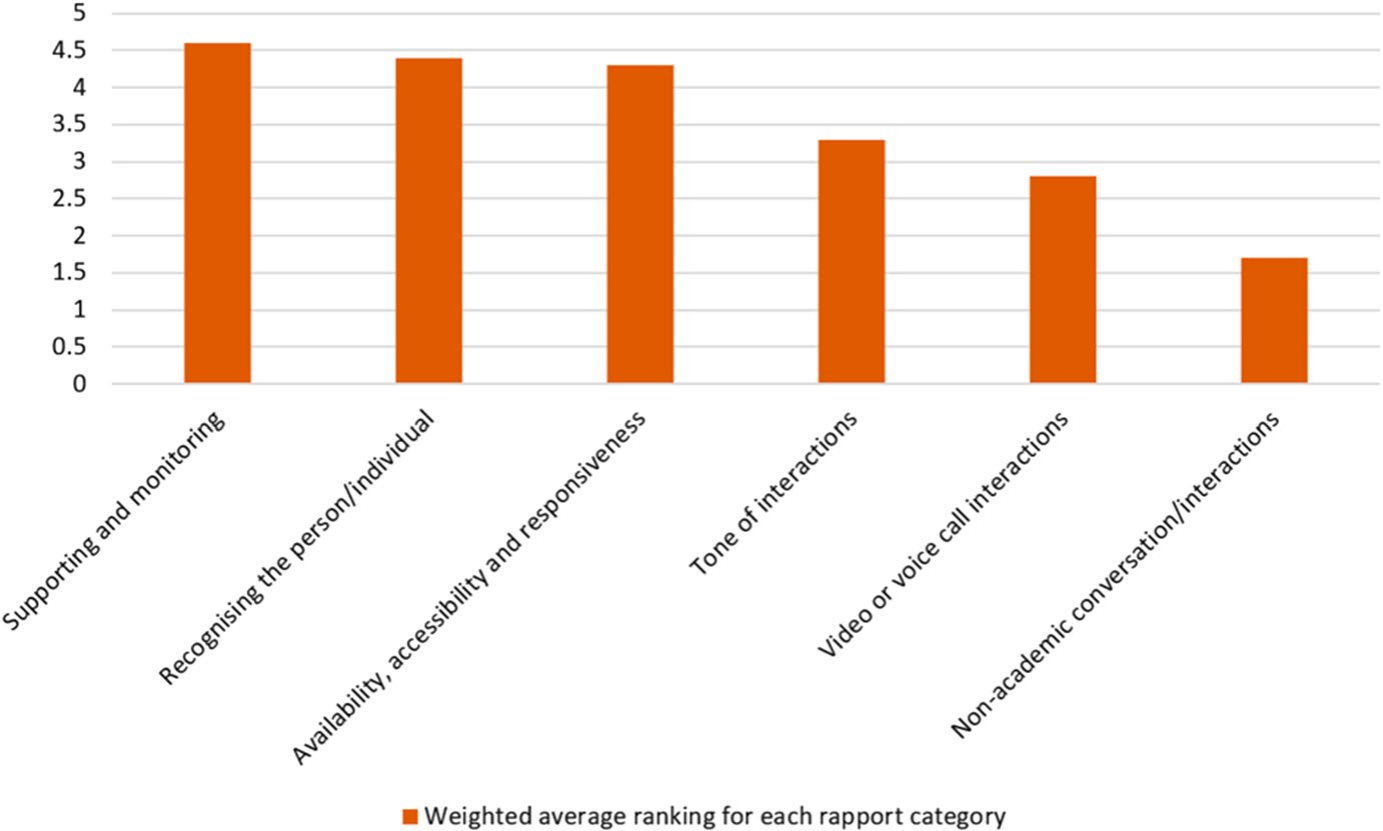
Weighted average ranking for rapport categories (6—most important, 1—least important) (Q14)

Question 14 contained a further six categories of rapport drawn from teacher interviews in Murphy and Rodríguez-Manzanares’ 2012 study which relate specifically to distance education. Participants ranked these categories in order of importance to them when teaching EAP online, to include both synchronous and asynchronous teaching and learning activities. Figure 3 shows the distribution of responses (marginal frequency) given for the most important and least important of these rapport categories when teaching online and Fig. 4 shows the weighted average ranking.

**Fig. 5:**
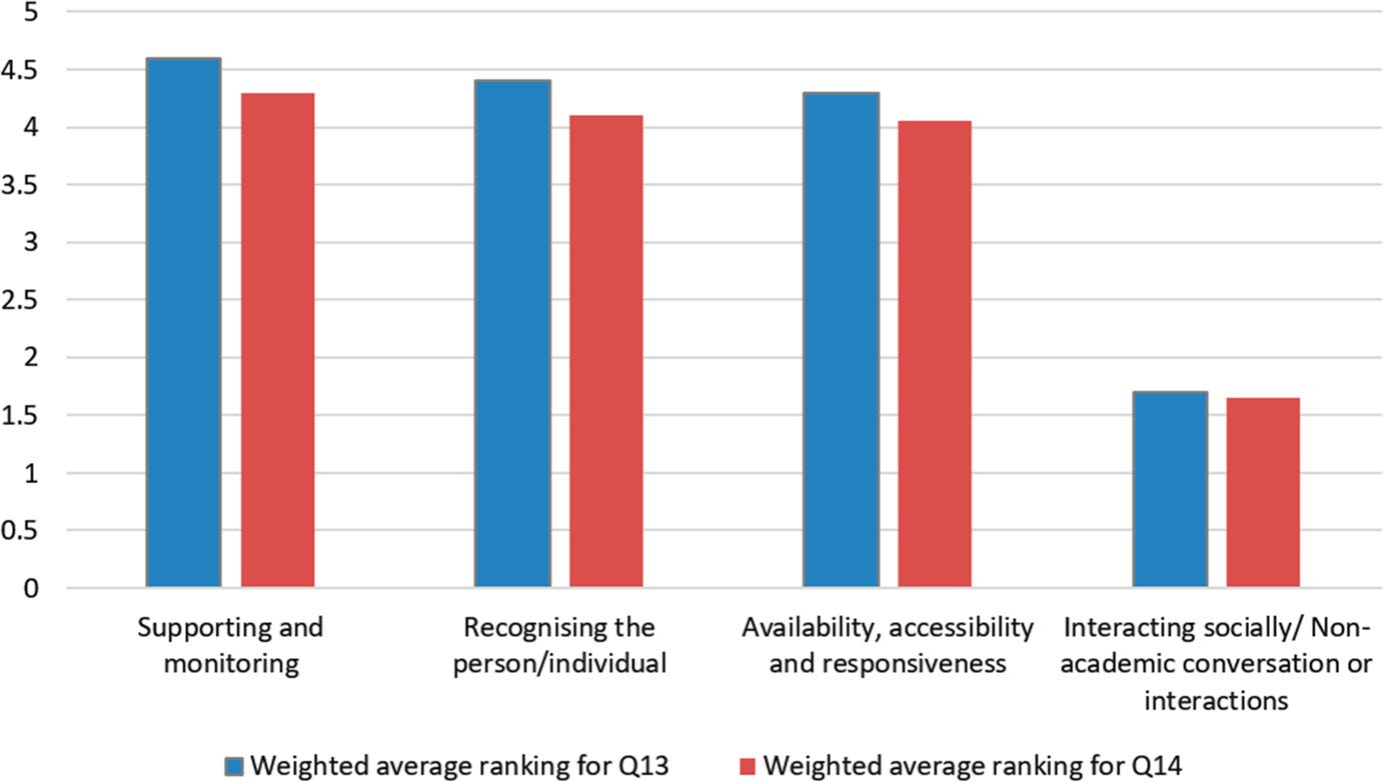
Weighted average ranking for rapport categories (6—most important, 1—least important) (Q13–14)

The eight categories of rapport featured in question 13 were synthesised by Murphy and Rodríguez-Manzanares’ after reviewing both distance education literature and the literature in general (2012). The six categories of rapport featured in question 14 were identified by the same researchers in their own 2012 study and relate specifically to distance education. There is an overlap of four categories of rapport between these two sets of categories. These are listed in Fig. 5 alongside a comparison of the weighted average rankings of these four categories. The data were adjusted by ratio so that both weighted average rankings use the same scale (6 being the most important when teaching online and 1 being the least important to respondents). The positive correlation in the data set trends from questions 13 and 14 indicates reliable and valid results.

This data set answers RQ1 by giving insights into EAP teachers’ perceptions of the importance of various rapport categories when building teacher–student rapport in synchronous and asynchronous OLEs. The results from Figs. 1, 2, 3, 4, and 5 indicate that the most important rapport categories were:

- Supporting and monitoring
- Recognising the person/individual
- Availability, accessibility and responsiveness
- Communicating effectively

The least important rapport categories were:

- Interacting socially/non-academic conversation or interactions
- Sharing, mirroring, mimicking and matching
- Video or voice call interactions

#### Questionnaire section three—quantitative data

**Fig. 6:**
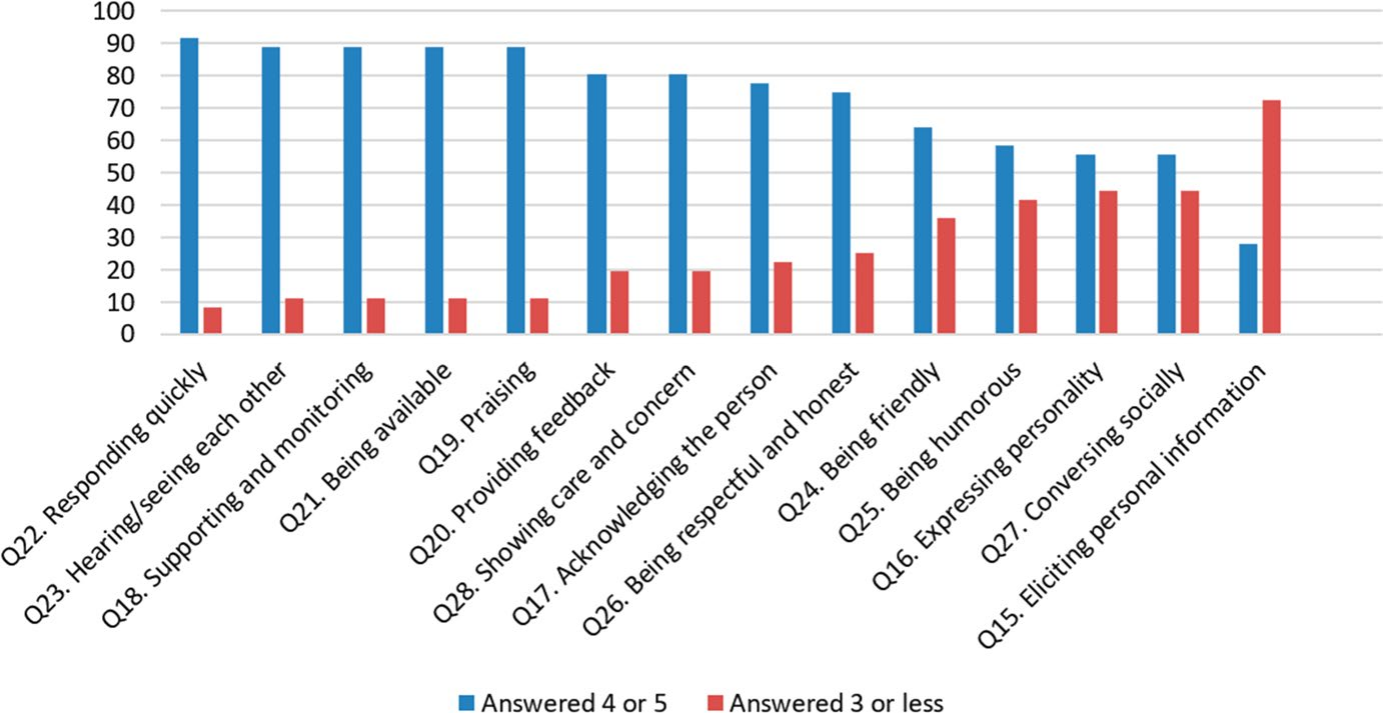
Distribution of positive (value 4–5) and neutral/negative (< 3) responses in section three

The quantitative data collected in section three of the questionnaire correspond directly to RQ3 (*Which online teaching practices and student-directed rapport behaviours do teachers employ to help develop teacher–student rapport online?*)*.* Figure 6 shows the distribution of responses over the fourteen questions in section three. Answers given at 4 or 5 show that respondents positively employed the specified online teaching practices and student-directed rapport behaviours. Answers given at 3 or below show that teachers chose not to employ these methods of rapport building online or experienced challenges doing so. For thirteen out of the fourteen sub-categories of rapport, respondents did employ online teaching practices and student-directed rapport behaviours more than they did not (or were not able to).

**Fig. 7:**
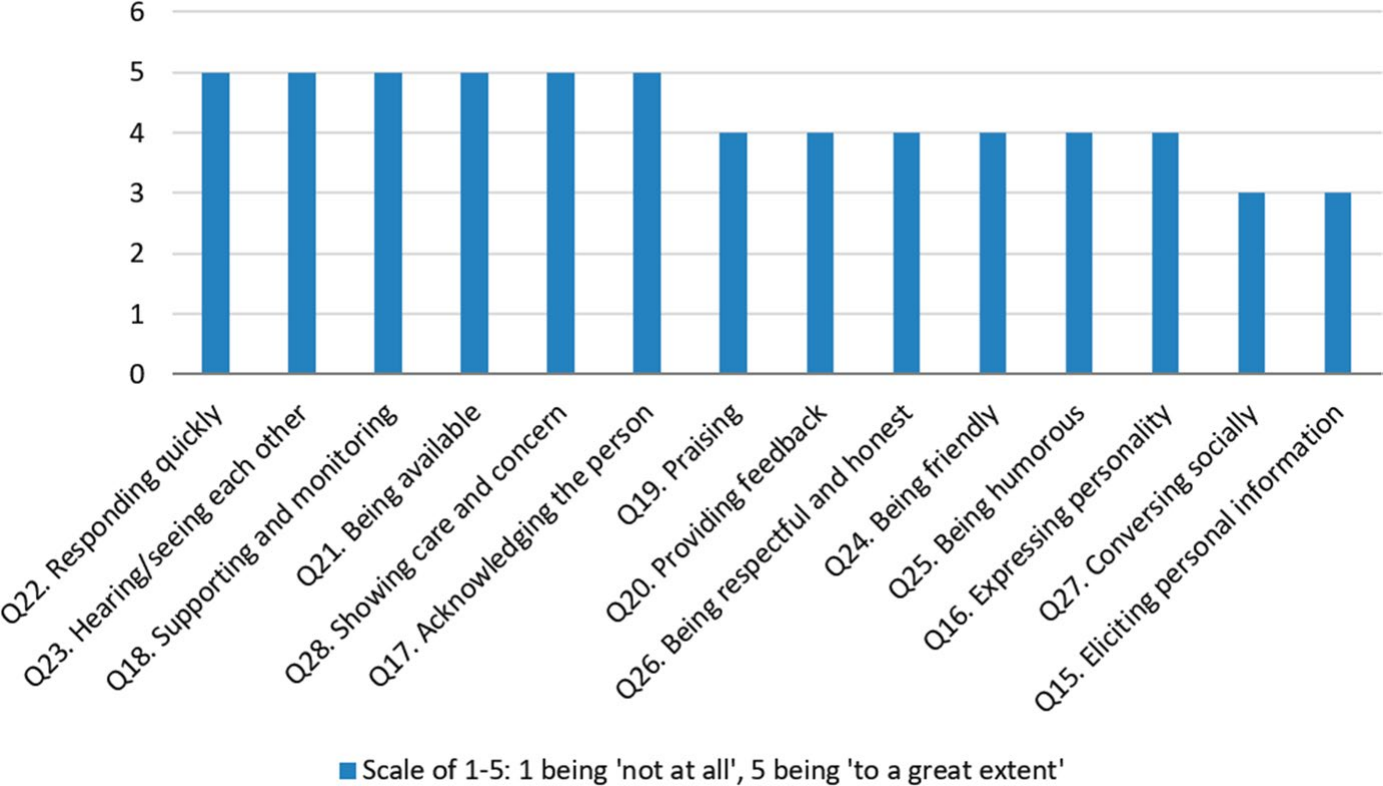
The mode of the frequency distribution of responses to questions in section three

Figure 7 shows the mode of the frequency distribution of responses to the questions in section three (the most frequently observed data value). This confirms the data in Fig. 6 since the data trend is comparable.

Analysis of data from both Figs. 6 and 7 answers RQ3 by indicating that the online teaching practices and student-directed rapport behaviours which teachers employed the most to help develop TSR were:

- Responding quickly
- Hearing/seeing each other
- Supporting and monitoring
- Being available

Those teaching practices and behaviours employed the least to develop TSR were:

- Conversing socially
- Eliciting personal information

### Phase 1 and 2 qualitative findings and overall discussion

#### Introduction

The qualitative data from the Phase 1 typed-responses and the Phase 2 interviews answer RQ2 (*What perceived constraints and challenges were reported by teachers when building teacher–student rapport online?)* and RQ3 (*Which online teaching practices and student-directed rapport behaviours did teachers employ to help develop teacher–student rapport online?)*. Following detailed analysis and coding of these combined qualitative data six major themes were identified:

1. Time constraints.
2. Online communication constraints in online sessions.
3. Teacher–student communication outside online sessions.
4. Teacher availability and accessibility.
5. Technology and digital skills constraints.
6. Teacher beliefs about rapport.

Due to the data set’s size and the limitations of this work not all data or themes can be discussed here. Three qualitative data themes were selected to be discussed in this section (alongside implications and recommendations) on the basis of their correlation with the quantitative results from Phase 1 (Table 3) since this indicates their saliency. These findings are discussed below and directly correspond to RQ2 and RQ3 whilst also referencing the quantitative findings from RQ1 and relevant literature.

**Table 3:** Correlation of Phase 1 and Phase 2 data

Phase 1 Quantitative data for RQ1 Most important rapport categories according to teachers	Phase 1 Quantitative data for RQ3 Most practised student-directed rapport behaviours by teachers	Phase 2 Associated qualitative data themes
Supporting and monitoring Recognising the person/individual	Supporting and monitoring	Online communication in online sessions Teacher–student communication outside online sessions
Availability, accessibility and responsiveness	Responding quickly Being available	Teacher availability and accessibility
Communicating effectively	Hearing/seeing each other	Online communication in online sessions Teacher–student communication outside online sessions

#### Challenges and constraints (RQ2)

The challenges and constraints which most significantly affected teacher–student rapport have been summarised in Table 4 for the selected themes. These factors have been expanded upon below with teacher quotations for clarity in order to answer RQ2. T1, T2 and T3 refer to the three teacher interviewees and TQ refers to an anonymous teacher from the qualitative questionnaire data.

**Table 4:** Summary of qualitative data for the three selected themes

Online communication in online sessions	Teacher–student communication outside online sessions	Teacher availability and accessibility
Lack of participation, communication or response from students	Use of pre- and post- online session time	Lack of parameters and guidance for teachers
Decrease in informal teacher–student interactions	Lack of guidance for teachers and students	Lack of parameters and guidance for students

##### Online communication in online sessions

This theme was the most significant overall in terms of the amount of data collected from teachers about the perceived challenges which inhibited effective teacher–student communication in the OLE and thus negatively affected TSR. The main reported challenges are listed in Table 4 and should be viewed in the light of Phase 1 data which showed that ‘Communicating effectively’ was one of the most important rapport-building categories for teachers.

It was noted that not all students turned on their cameras in online sessions, nor did all students participate or contribute via the chat bar or verbally resulting in a “*wall of silence*” (TQ) and “*depersonalised”* interactions (TQ) which impeded teachers’ perceived efforts to foster rapport. One teacher referred to this as “*talking into the void*” (TQ), a now-familiar expression (Aebersold 2021). Several teachers reported feeling uncomfortable with one-way visual communication which echo reports in the literature where both teacher–student relationships and teaching experiences in the online environment are described in terms of disconnection (Weller 2020; Wilkins 2014) and do not reflect the “mutual attention” and “easy communication” necessary for rapport building (Hall et al. 2009, p. 324). Interestingly, despite these communication challenges, teachers reported ‘Hearing/seeing each other’ as one of their most employed rapport-building behaviours in the OLE in Phase 1 data which indicates the effort teachers made during the study focus period.

These findings build on existing evidence that the absence of visual presence and nonverbal communication negatively affects instructional immediacy and TSR (Murphy and Rodríguez-Manzanares 2012). This is significant since the literature reports a lack of nonverbal behaviours as particularly problematic for international students’ cross-cultural communication development (Wang and Yang 2019), and that cross-cultural adjustment can affect international students’ academic performance (Misra et al. 2003).

Teachers speculated that a lack of student participation or activated cameras may result from students’ personal choice, privacy concerns, lack of functioning equipment, personality issues such as shyness, time pressure, cultural reasons or lack of engagement. There is, therefore, a clear need for teachers to identify the reasons behind this behaviour and to address these issues directly as they would in a F2F classroom with non-participating students. Another implication here is whether there should be institutional requirements for students to activate cameras during online sessions or whether this impedes on students’ privacy or cultural needs. Smaller online classes could provide students with the opportunity to build rapport and trust with their peers which in turn may enable confidence and engagement sufficient for camera and microphone activation.

Teachers reported being unable to interact with students in the OLE in the same way they would in the F2F environment. The reported increase in teacher-talking-time in online sessions may have contributed to this perceived decrease in informal teacher–student interactions. Several teachers reported difficulties with praising, using humour and being spontaneous. Teachers also expressed a perception that the nature of communication in the OLE impaired their ability to express friendliness or warmth (“*I… ‘fell by the wayside’ of my warmth, which would not happen in the classroom*”—TQ). These findings correlate with Phase 1 findings where teachers did not employ or had challenges employing the rapport category of ‘Being friendly’ (36.1% of teachers) and ‘Being humorous’ (41.6% of teachers). These not only give new insights into building TSR in the OLE but also carry implications in the light of Krishnan and Vrcelj’s study (2009) which showed that a friendly class atmosphere is one of the most important factors affecting international students’ learning. These findings also support Bailenson (2021) in that technical limitations and limited social-context cues negatively impact online interaction.

Since communication issues impede TSR in online sessions then opportunities for more natural and individualised teacher–student interaction could be sourced elsewhere in the OLE, for example in pre- or post-online session conversations or in 1–1 tutorials. An alternative solution could be pre-empting a lack of student communication in the OLE. Comprehensive online student inductions could include materials to set student learning expectations; induction activities in which the benefits of fully participating online could be presented; and the rationale for different online learning activities could be shared with students. This may more effectively induct students into UK HE culture online while serving as a strong foundation for TSR and its associated benefits. Teachers could set clear expectations with students at the beginning of the course about student participation and behaviour in class, to include when cameras are required, since the literature states that teacher–student relationships are strengthened when students’ learning expectations are met (Estepp and Roberts 2015).

##### Teacher–student communication outside online sessions

The main challenges related to building TSR within the theme of teacher–student communication outside online sessions were the use of pre- and post-online session time and a lack of guidance for both teachers and students.

Several teachers reported that they were able to use pre- and post-online session time to interact socially and academically with students while others were not able to do so. TQ said that this time allowed conscious rapport building with students: “<*they*> *seem to really like these moments….they also ask personal questions too in these periods, which is fine for me*” (TQ). Other teachers, however, were not able to use this time for student interaction due to their workload. T1 stated that a “*flexible rollover*” window between online classes would have given a “*buffer time”* for this if class timetabling did not currently permit such interactions. A few teachers, however, reported that their own introversion was an inhibitor of interaction online with students, and one stated: “*There was some chit chat with students before class, but it's often forced rather than natural*” (TQ). This confirms previous research giving social interaction difficulties as one of the major challenges of building TSR in the OLE (Murphy and Rodríguez-Manzanares 2012; Rapanta et al. 2020).

It seems that dedicated opportunities for building TSR through informal teacher–student communication could be integrated around online sessions by utilising pre- and post-online session time if timetabling and teacher workload were adjusted to support this. Time spent on content delivery would therefore not be affected and learning outcomes could still be achieved alongside the conscious development of TSR. In reality, since EAP courses are intensive and content-heavy, institutional constraints simply may not permit this.

##### Teacher availability and accessibility

A key factor in TSR is how available or accessible teachers are since this directly impacts on students’ perceptions of teacher presence in the OLE. High teacher presence increases student motivation, engagement, satisfaction and thus learning (Baker 2010). The main challenge related to building TSR within the theme of teacher availability and accessibility was a lack of guidance in this area for both teachers and students.

Firstly, several teachers reported that a lack of clear guidelines from their institution manifested in different working hours for teachers and inconsistent working practices across cohorts of teachers, which was compounded by differences in teacher–student and teacher–teacher time zones, and difficulties working from home (for example balancing work and childcare): “*Most of the time I responded immediately, but I feel that it's very important that this does not become an expectation”* (TQ). Multiple teachers responded to students outside their working hours. A strong theme emerging from teachers was the need for clear guidelines and expectations of the appropriate response time to student communications such as emails: “*I think you need to be quite clear with [students] when you're available and how they should be contacting you*…*No-one, from my experience, was quite sure what those parameters should be”* (T1). Several teachers referred to the negative effect on student autonomy when teachers were perceived to be too accessible and available: “*Responding instantly to student messages….would be detrimental to teachers' wellbeing and give students unrealistic expectations when going on to their master's courses*” (TQ).

Secondly, regarding students, a few teachers expressed concerns that students might not be contacting them outside the online session because of a lack of professional guidelines for doing so appropriately. A lack of enforceable rules for student–teacher communication also resulted in some students over-using Teams chat messaging or other platforms.

Despite these challenges, ‘Responding quickly’ and ‘Being available’ emerged as two of the most employed rapport-building behaviours for teachers from the quantitative data while ‘Availability, accessibility and responsiveness’ was one of the most important. It is clear, therefore, that teachers see value in this rapport behaviour. There seems a need for clarity from institutions on when it is appropriate for students to contact their teachers, in what time frame teachers are expected to reply and what platforms or technology these interactions should use in order to ensure a uniform approach to teacher–student communication. Such policies could be communicated to both teachers and students during course induction and may reduce teacher workload, improve quality assurance and allow for the evaluation or monitoring of teacher performance while reducing missed opportunities for TSR development. From a student perspective, the literature is clear that international EAP students need support with socio-cultural and psychological adaptation to studying in the HE context (Berry 1997), which guidelines on appropriate teacher–student communication would provide. Ideally, online teacher availability and accessibility must fit within institutional constraints, those of teacher workloads and realistic student needs. It also needs to support EAP students’ development of autonomy while still allowing for TSR development.

#### Developing TSR online (RQ3)

RQ3 sought to uncover which online teaching practices and student-directed rapport behaviours teachers employed to help develop teacher–student rapport online. Phase 1 quantitative data showed that teachers did employ these practices and behaviours more than they did not (or were not able to) for thirteen out of the fourteen sub-categories of rapport. It is therefore clear that, despite the challenges of building TSR, EAP teachers in this study did actively participate in its development in the OLE.

The most significant theme for RQ3 was that many teachers focused on interactions with their students in 1–1 tutorials as TSR opportunities since they more closely reflected those in the F2F environment. It was suggested by several teachers that it was easier to build TSR during 1–1 student tutorials than in online sessions (“*I got to know them *via* tutorials not primarily through class teaching”*—TQ). Group tutorials were not judged to be as effective for building TSR in comparison with 1–1 tutorials, perhaps due to the fact that they shared the same communication challenges as online sessions with multiple students (“*[you can] help [to] build rapport then, in a synchronous tutorial, more so than you could in a group taught class*”—T1). One teacher reported that “*the amount of help and support for students is limited by the course constraints (they get one personal tutorial per week*)” (TQ). This implies that a greater number of weekly tutorials could provide more student support and thus more opportunities for TSR development if institutional constraints would allow for these. ‘Supporting and monitoring’ was chosen as the most important rapport category and one of the most employed rapport-building behaviours which indicates it is a key factor for teachers.

According to the data, the effectiveness and ease of actioning rapport behaviours seems to vary according to the number of students in the interactions. The findings from this study confirm that all six of Murphy and Rodríguez-Manzanares’ (2012) categories of rapport in DE (Table 1) are perceived to be easier to employ in 1–1 interactions with students and are therefore perceived as more effective (either asynchronously such as via emails or synchronously such as in tutorials) than in online sessions. Further literature states that rapport is dynamic and evolving, requiring the participation and collaboration of both teacher and individual student (Rogers 2012). It is not surprising, therefore, that teachers reported challenges in building rapport with students in online sessions since rapport is only experienced via dyadic interactions (Altman 1990) which are necessarily limited in group classes and plentiful in 1–1 or small group interactions.

While rapport appears to be more easily fostered in 1–1 interactions, teachers still reported employing a variety of actions or behaviours to build rapport in the live online sessions. Those which fall within the sub-categories of rapport in Table 1 (Murphy and Rodríguez-Manzanares 2012) will not be discussed here, but novel approaches will. These include trying to find a shared context with students to facilitate conversation. Several teachers mentioned learning personal information or characteristics about their students from live online session conversations and referring to these in later classes. A variety of chat-bar and audio-visual strategies were employed by teachers in these sessions to build rapport and encourage teacher–student interaction, for example using the whiteboard tool or polls to encourage student responses. Teachers also nominated students directly for a verbal response or chat bar comment. Multiple teachers report using the status update feature on the VLE Blackboard Collaborate (which students can use to indicate their mood); using a ‘thumbs up’ sign for agreement; or using other emojis in order to convey attitudes, feelings and responses quickly. At the beginning of online sessions, a few teachers reported asking students to enter an emoji into the chat bar to symbolise their mood or feelings. This was then used as a springboard for an informal chat and to lead into the lesson. These findings support those of Murphy and Rodríguez-Manzanares’ studies (2008a, b) which concluded that teachers engage in more deliberate rapport-building behaviours and strategies in the OLE as compensation for a lack of physical presence. These findings also correspond with Phase 1 data in that ‘Supporting and monitoring’ was one of the most employed rapport building behaviours and ‘Communicating effectively’ was one of the most important to teachers. The implication here is that, while it is universally acknowledged that building TSR in the online sessions is challenging, teachers perceive that much can still be done and thus online sessions should not be completely disregarded as a space where TSR can be built.

## Conclusion

This study sought to explore EAP teachers’ experiences of building rapport with international students in the novel online learning environment in the early Covid-19 era via quantitative and qualitative analyses of teacher perceptions. Overall conclusions and associated recommendations are summarised here.

Based on quantitative and qualitative analysis of teacher perceptions about their experiences, it is clear that most teachers valued rapport-building and made concerted efforts to do so in the OLE despite the manifold challenges they experienced. Other teachers appear to have been more limited in the range of rapport-building behaviours they employed due to their personal beliefs about TSR or a perceived lack of institutional support. This study therefore indicates a need for clearer and more explicit institutional guidelines to teachers about the particular importance of rapport-building in the OLE and how this can be achieved, as well as defined timeframes and methods for responding to student communication.

Second, the findings indicate that students may require extra support in their induction to UK HE culture online to include the importance of communicating with their teacher, as well as practical guidelines on the appropriate times and methods for doing so. Setting clear expectations of student behaviour and participation in the online space may mitigate some of the identified challenges with building TSR.

Thirdly, these findings support those from Murphy and Rodríguez-Manzanares’ study (2012) which concluded that teacher–student rapport-building is particularly necessary in distance education (and, by association, online education) due to the absence of F2F communication, and that online TSR requires more effort to build. It appears more difficult to build TSR in group settings in comparison to 1–1 tutorials or small groups even if cameras are activated. This study suggests online sessions do not carry appropriate opportunities for effective teacher–student rapport building so alternative conscious TSR-building opportunities are necessary in the OLE. If live online sessions could primarily be used as opportunities to meet learning outcomes, then the necessary and conscious task of building TSR might be more effective in frequent 1–1 tutorials with students or at least during pre- and post-online session time if institutional constraints permitted.

Future research could address the multiple independent variables which may affect how effectively rapport is established online or F2F, between teachers and students, or students and their peers. For international students, such variables could include culture and English language level.

## Abbreviations

1-1: One-to-one (individual)
EAP: English for Academic Purposes
F2F: Face-to-face
HE: Higher education
OLE: Online learning environment
Q: Question
RQ1: Research sub-question 1
RQ2: Research sub-question 2
RQ3: Research sub-question 3
T1: Teacher 1 (interview participant)
T2: Teacher 2 (interview participant)
T3: Teacher 3 (interview participant)
TQ: Teacher (anonymous teacher from qualitative questionnaire data)
TSR: Teacher–student rapport
VLE: Virtual learning environment
